# The Effect of Manuka Honey on dHL-60 Cytokine, Chemokine, and Matrix-Degrading Enzyme Release under Inflammatory Conditions

**DOI:** 10.20900/mo.20190005

**Published:** 2019-04-25

**Authors:** Benjamin A. Minden-Birkenmaier, Meghan B. Meadows, Kasyap Cherukuri, Matthew P. Smeltzer, Richard A. Smith, Marko Z. Radic, Gary L. Bowlin

**Affiliations:** 1 Department of Biomedical Engineering, University of Memphis, 330 Engineering Technology Building, Memphis, TN 38152, USA; 2 Division of Epidemiology, Biostatistics, and Environmental Health, School of Public Health, University of Memphis, 222 Robison Hall, Memphis, TN 38152, USA; 3 Department of Orthopaedic Surgery & Biomedical Engineering, University of Tennessee Health Science Center, E228A Coleman Building, 956 Court Avenue, Memphis, TN 38163, USA; 4 Department of Microbiology, Immunology and Biochemistry, University of Tennessee Health Science Center, 201 Molecular Science Building, 858 Madison Ave., Memphis, TN 38152, USA

**Keywords:** Manuka honey, inflammation, neutrophil, HL-60, cytokine

## Abstract

A large body of *in vivo* and *in vitro* evidence indicates that Manuka honey resolves inflammation and promotes healing when applied topically to a wound. In this study, the effect of two different concentrations (0.5% and 3% v/v) of Manuka honey on the release of cytokines, chemokines, and matrix-degrading enzymes from neutrophils was examined using a differentiated HL-60 cell line model in the presence of inflammatory stimuli. The results indicate that 0.5% honey decreased TNF-α, IL-1β, MIP-1α, MIP-1β, IL-12 p70, MMP-9, MMP-1, FGF-13, IL-1ra, and IL-4 release, but increased MIP-3α, Proteinase 3, VEGF, and IL-8 levels. In contrast, 3% honey reduced the release of all analytes except TNF-α, whose release was increased. Together, these results demonstrate a dose-dependent ability of Manuka honey to modify the release of cytokines, chemokines, and matrix-degrading enzymes that promote or inhibit inflammation and/or healing within a wound. The findings of this study provide further guidance for the future use of Manuka honey in wounds or tissue engineering templates. Future *in vivo* investigation is warranted to validate the *in vitro* results and translate these results to physiologically relevant environments.

## INTRODUCTION

Neutrophils, the first-responding leukocytes which enter a wound site soon after injury, orchestrate the initiation, amplification, and resolution of the inflammatory response. In addition to their role as phagocytes, in which they engulf and destroy invading bacteria, neutrophils release a host of molecular agents which affect the inflammatory state of the wound. In the presence of bacterial signals such as LPS and fMLP or native inflammatory signals such as IFN-γ or GM-CSF, neutrophils become pro-inflammatory, releasing superoxide and proteases which degrade bacteria and native ECM, recruiting more immune cells, and perpetuating the cycle of inflammation [1–4]. In the presence of anti-inflammatory signals such as TGF-β or IL-4, neutrophils attenuate their superoxide and protease release and instead release immunosuppressive agents such as IL-1ra [5–8]. An initial pro-inflammatory response is beneficial in that it kills bacteria, frees vascular endothelial cells for angiogenesis, and recruits a host of T-cells, eosinophils, basophils, monocytes, and NK cells to continue fighting infection [3,8–10]. However, persistent, non-resolving inflammatory neutrophil activity has been implicated in chronic inflammation that impedes wound healing and causes sepsis [11–13]. Additionally, this activity has been linked to inflammatory diseases such as rheumatoid arthritis and atherosclerosis [14–16]. It is necessary to develop and optimize treatments to shift neutrophils from this inflammatory state to an anti-inflammatory state to resolve such pathological inflammation.

Manuka honey, a variety of honey produced from the nectar of the New Zealand shrub *Leptospermum scoparium*, has demonstrated remarkable wound healing properties. The high sugar concentration of Manuka honey creates an osmotic gradient when placed on a wound, drawing fluid and nutrients from deeper tissue up through the wound and flushing bacteria and debris from the site [17,18]. Flavonoids derived from the honey’s floral sources scavenge free radicals, reducing tissue damage, while the low pH of the honey increases fibroblast and macrophage activity and helps oxygenate the wound [17,19,20]. Previous work by Alvarez-Suarez *et al.* has quantified this phenolic content of Manuka honey via HPLC-MS, and the authors theorized that these components improve the intracellular antioxidant response [20]. The honey also protects the wound site, creating a viscous layer at the top which impedes additional bacterial deposition and keeps the wound hydrated, and the high sugar content provides a glucose source for proliferating fibroblasts and endothelial cells in the area [18]. Manuka honey kills bacteria via its osmotic potential and the presence of methylglyoxal [21–25]. *In vitro* testing has indicated the effectiveness of Manuka honey against a variety of bacteria, including antibiotic-resistant bacteria such as MRSA [21,26–28]. These protective and pro-healing effects of Manuka honey in wounds have been demonstrated both in animal models and clinical trials [29–37]. However, some animal models and *in vitro* testing have indicated that high concentrations of Manuka honey can have a deleterious cytotoxic effect [38–40]. These findings indicate that research should focus on concentrations of Manuka honey that are low enough to avoid this cytotoxic effect.

In this study, a dHL-60 model of a neutrophil is used. The HL-60 cell line can be differentiated to a neutrophil-like phenotype using DMSO in a procedure that has been well-optimized [41]. Because neutrophil cytokine release can vary from donor to donor, it was decided to use this standardized cell model for greater reproducibility [42]. Release levels have been compared between dHL-60s and human peripheral blood neutrophils of a number of cytokines and chemokines, including TNF-α, IL-1β, IL-6, IL-12, CXCL8/IL-8, CCL2/MCP-1, CCL3/MIP-1α, CCL4/MIP-1β, and CCL5/RANTES. Release was compared under control and LPS-stimulated conditions, and the study found that although minor quantitative differences existed in the levels of some of these cytokines, the overall cytokine response was qualitatively similar between the two cell types at each condition [8]. This differentiated cell line model has become a useful tool to study neutrophil cytokine/chemokine release [43,44].

We have previously reported several effects of Manuka honey on this dHL-60 model, including reducing superoxide release, chemotaxis, and activation of the inflammatory transcription factor NF-κB. Additionally, we found that levels of Manuka honey at 5% v/v or above were cytotoxic to the dHL-60s, in line with what previous studies have reported in other cell types [38,45]. As such, in this effort, we focus on concentrations of honey below this cytotoxic limit.

This study investigates the effect of Manuka honey on cytokine, chemokine, and matrix-degrading enzyme release of the dHL-60 model under pro-inflammatory stimuli. Four different stimulating factors (2 exogenous and 2 endogenous) were used. The bacterial product fMLP acts on a GCPR to initiate the β-arrestin pathway, the PLC β 2/3 pathway, and the PI3-kinase pathway to induce actin reorganization, NADPH oxidase activity, and granule release [46,47]. LPS, a bacterial outer membrane component, acts on the TLR4 to initiate the MYD88 pathway, increasing production of a variety of inflammatory cytokines for release [48]. GM-CSF binds a cytokine receptor and acts through the JAK/STAT pathway, PI3K pathway, and SHC pathways to prime neutrophil oxidative metabolism and cytokine expression [49–51]. IFN-γ similarly binds its cytokine receptor and acts through the JAK/STAT pathway to increase inflammatory cytokine expression [52,53]. The dHL-60s were cultured in the presence of these stimulators by themselves and in combination for 3 or 24 hours, and their supernatant was assayed for the pro-inflammatory signals TNF-α, IL-1β, CCL5/RANTES, and IL-12 p70, the anti-inflammatory signals IL-1ra and IL-4, the matrix reorganization enzymes MMP-1, MMP-9, and Proteinase 3, the growth factors FGF-13 and VEGF, and the chemoattractants CXCL8/IL-8, CCL3/MIP-1α, CCL4/MIP-1β, CCL2/MCP-1, and CCL20/MIP-3α. The experiments were repeated in the presence of 0.5% v/v and 3% v/v Manuka honey to ascertain the effect of Manuka honey on cytokine, chemokine, and matrix-degrading enzyme release. These experiments will inform the greater understanding of how Manuka honey affects neutrophils within a wound site, and how those effects can be harnessed as a biomaterial additive.

## MATERIALS AND METHODS

### HL-60 Culture and Differentiation

HL-60 cells purchased from the American Type Culture Collection (ATCC, CCL240) (Manasses, VA, USA) were cultured at a cell density range of 2 × 10^5^ to 1 × 10^6^ cells per millilitre in RPMI (Hyclone, Logan, UT, USA) with 10% v/v non-heat-inactivated FBS, 1% v/v Pen/Strep, and 1% _L_-glutamine (hereafter referred to as culture medium), all purchased from Hyclone (Logan, UT, USA). Cells were grown at 37 °C in a 5% CO_2_ incubator in T-25 and T-75 culture flasks (Thermo Scientific, Rochester, NY, USA). During culture, medium was changed every 3–4 days and cells were passaged when cell density reached 5 × 10^5^ cells/mL. Cells were used for passage numbers up to 30. Differentiation to a neutrophil-like phenotype was accomplished using a procedure validated in previous studies [54,55]. Briefly, cells were differentiated by adding 1.25% DMSO (Sigma Aldrich, St. Louis, MO, USA) to the culture medium for six days, replenishing medium/DMSO on the third day. Differentiation was confirmed morphologically by permeabilizing with 0.17 mM Triton X-100 (Fisher Scientific, Hampton, NH, USA) for 5 min, then fixing in 10% buffered formalin (Fisher Scientific, Hampton, NH, USA) and staining with DAPI (NucBlue Fixed Cell Stain ReadyProbes reagent) for 5 min at stock concentration and phalloidin-conjugated Alexa Fluor 488 (ActinGreen 488 Ready Probes reagent) (both from Invitrogen, Carlsbad, CA, USA) for 30 min also at stock concentration according to the manufacturer’s protocols. Cells were imaged with an Olympus microscope (model BX34F) with an attached Olympus DP73 digital color camera and Olympus U-HGLGPS fluorescent light source (Olympus, Shinjuku, Tokyo, Japan). The percentage of differentiated cells (kidney-shaped nucleus) was calculated to be 69%, comparable to the percentage reported in literature [54,56].

### Cytokine Release Experiment

To initiate the experiments, dHL-60s were seeded in a 96 well plate at 400,000 cells per well (8 million cells/mL) in 150 μL of culture media with either no additives (control), 1 μg/mL LPS, 10^−7^ M fMLP, 100 U/mL GM-CSF, 100 U/mL IFN-γ, both 1 μg/mL LPS and 10^−7^ M fMLP, both 1 μg/mL LPS and 100 U/mL IFN-γ, or all four of 1 μg/mL LPS, 10^−7^ M fMLP, 100 U/mL GM-CSF, and 100 U/mL IFN-γ (LPS from InvivoGen, San Diego, CA, USA; fMLP from Sigma Aldrich, St. Louis, MO, USA; IFN-γ and GM-CSF from R&D Systems, Minneapolis, MN, USA). Concentrations of these analytes were taken from prior studies. 100 U/mL GM-CSF has been demonstrated to optimize TLR2 and CD14 expression and IL-8 release in neutrophils [50]. Similarly, 100 U/mL IFN-γ has been demonstrated to maximize MIP-1α and MIP-1β release when used in conjunction with LPS [57]. The combination of 1 μg/mL LPS and 10^−7^ M fMLP maximizes neutrophil superoxide output [58]. Cells were cultured for 3 or 24 hours, and then centrifuged to remove and save supernatant. This supernatant was analyzed using a multiplexed magnetic bead immunoassay (R&D Systems, Minneapolis, MN, USA) on a MAGPIX® reader (Luminex, Austin, TX, USA). These results were analyzed to determine the stimuli and combinations which elicited the greatest overall release response. Based on these results, it was decided to focus on the groups stimulated with LPS, LPS and fMLP, and all four stimuli (LPS, fMLP, IFN-γ, and GM-CSF). The experiment was then repeated with these stimulation groups in the presence of 0.5% and 3% Manuka honey (UMF 12+, Manuka Guard, Monterey, CA, USA) alongside honey/media blanks which contained no cells. Honey was added at the same time as the stimulus (timepoint 0). These supernatants were assayed using the MAGPIX® immunoassay. Additionally, a control group and an LPS-stimulated group without honey were run again for 24 hours and their values were compared against the values from the first kit run to determine reproducibility. All supernatant samples were initially run using a dilution factor of 2 in assay diluent, and select samples whose readings were above the maximum level of detection (Max LOD) were re-run using a dilution factor of 200 in assay diluent.

### Statistical Analysis

Non-par ametric methods were used for comparisons due to the data distributions. The results of the control group and LPS-stimulated group measured in two subsequent assay runs were compared using a non-parametric alternative to the two-sample *t*-test, called the Wilcoxon rank sum test, for each analyte. Using an α = 0.05 value, no significant differences were found between the assay runs, indicating reproducibility and allowing comparisons to be made between the assay runs. Comparisons between stimulation groups at each honey level (0, 0.5%, and 3%) and between honey levels within stimulation groups were analyzed using the Kruskal Wallis test. The Bonferroni *p*-value adjustment was used to account for multiple comparisons (α = 0.05). Differences between timepoints within stimulation groups at each honey level were also tested using the Wilcoxon rank sum test (α = 0.05).

## RESULTS

**Figure 1.**
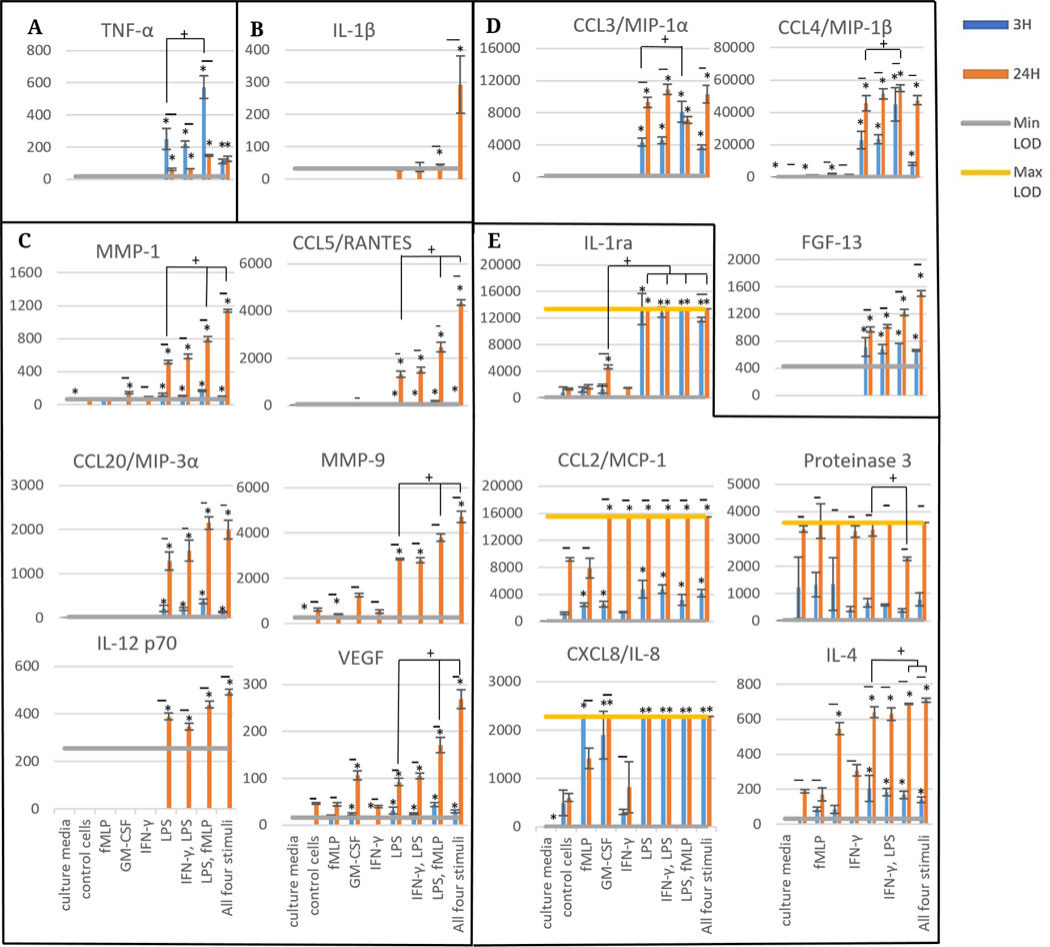
Released levels of various cytokines, chemokines, and matrix-degrading enzymes at 3 and 24 hours in pg/mL: Scale bars are mean ± standard deviation. “*” indicates a statistically significant difference from the non-stimulated control cells at that respective timepoint. No statistically significant differences were found between timepoints within treatment groups. Brackets with “+” indicate particular stimulation groups that are statistically significant from each other. A Kruskal-Wallis test was used with a Bonferroni adjustment for multiple comparisons, while a Wilcoxon rank sum test was used to establish significant differences between timepoints for each stimulation type (α = 0.05). Analytes are grouped by trend (**A**–**E**). *N* = 3.

The released levels of each analyte at 3 and 24 hours in the absence of honey are displayed in Figure 1. These figures are grouped by trend, and all individual *p* values for the comparisons shown in this paper are given in the Supplementary Materials. The differences mentioned in this Results section are statistically significant unless noted otherwise. The TNF-α data, grouped alone in Figure 1A, illustrates that TNF-α release is increased by LPS with a synergistic effect contributed by fMLP. TNF-α levels had a non-significant decreasing trend from 3 to 24 hours for the groups stimulated with LPS, IFN-γ and LPS, and LPS and fMLP. The results displayed in Figure 1B indicate that IL-1β release occurs only in the presence of all four stimuli (LPS, fMLP, IFN-γ and GM-CSF) and occurs after the first 3 hours post-stimulation. Figure 1C contains the release results for MMP-1, CCL5/RANTES, CCL20/MIP-3α, MMP-9, IL-12 p70, and VEGF. These analytes had minimal release at the 3-hour timepoint and an increasing trend from the LPS group to the LPS, fMLP group to the all four stimuli group (non-statistically significant in CCL20/MIP-3α and IL-12 p70). The MMP-1 and VEGF data indicate an increase in release in the GM-CSF-stimulated group relative to non-stimulated control, although this group is much lower than the LPS-stimulated group in the MMP-1 results. Together, these results suggest that of the stimuli used in this study, LPS is the most effective driver of release for these analytes, with fMLP providing a synergistic effect and the combination of all four stimuli providing an additional synergistic increase.

Figure 1D contains the CCL3/MIP-1α, CCL4/MIP-1β, and FGF-13 release data. These results indicate release at both 3 and 24 hours in the groups stimulated with LPS, IFN-γ and LPS, LPS and fMLP, and all four stimuli, with a non-significant increasing trend from 3 to 24 hours. CCL3/MIP-1α had an increase between the LPS and LPS, fMLP group at 3 hours, but a non-significant decrease between these groups at 24 hours. In contrast, the CCL4/MIP-1β results indicate an increasing trend between the LPS and LPS, fMLP groups at both 3 and 24 hours (not statistically significant at 3 hours). The FGF-13 results also reveal an increasing trend at 24 hours between the groups stimulated with LPS, LPS and fMLP, and all four stimuli (non-statistically significant). Similar to the results displayed in Figure 1A and 1C, these results suggest that LPS is the most effective of the stimuli at causing release of these analytes, with the addition of fMLP causing a synergistic response (not present at 24 hours in CCL3/MIP-1α). The use of all four stimuli caused an additional non-significant synergistic increase in FGF-13 release, but did not affect CCL3/MIP-1α release and caused a non-significant decreasing trend at 3 hours in CCL4/MIP-1β release.

Figure 1E contains the release results for IL-1ra, CCL2/MCP-1, Proteinase 3, CXCL8/IL-8, and IL-4. The Il-1ra, and IL-4 results indicate an increase in release at 24 hours in the GM-CSF group, less than the LPS-stimulated group for IL-1ra and for IL-4 at 3 hours but near the LPS group at 24 hours in IL-4. The release of IL-4 was greater in the LPS, fMLP and all four stimuli groups than the LPS group alone at 24 hours, while IL-1ra release was near to or exceeded the maximum level of detection (Max LOD) in all groups with LPS at both 3 and 24 hours. MCP-1 release exceeded the Max LOD in all groups except the culture media control, control cells, and fMLP at 24 hours, but at 3 hours had a non-significant increase in all LPS-stimulated groups relative to non-LPS groups. Proteinase 3 release likewise was close to or exceeded the Max LOD for all groups except LPS, fMLP and culture media at 24 hours, but at 3 hours had no apparent increase in any of the stimulation groups. Il-8 release approached or exceeded Max LOD for all groups except the IFN-γ group, culture media, control cells, fMLP at 24 hours, and GM-CSF at 3 hours. These results indicate that all stimuli except IFN-γ increase IL-8 release. Together, these results suggest that LPS is the most effective stimulus at causing IL-1ra, IL-4, and MCP-1 release, but has no effect on Proteinase 3 release and may not be more effective than fMLP or GM-CSF at causing IL-8 release.

**Figure 2.**
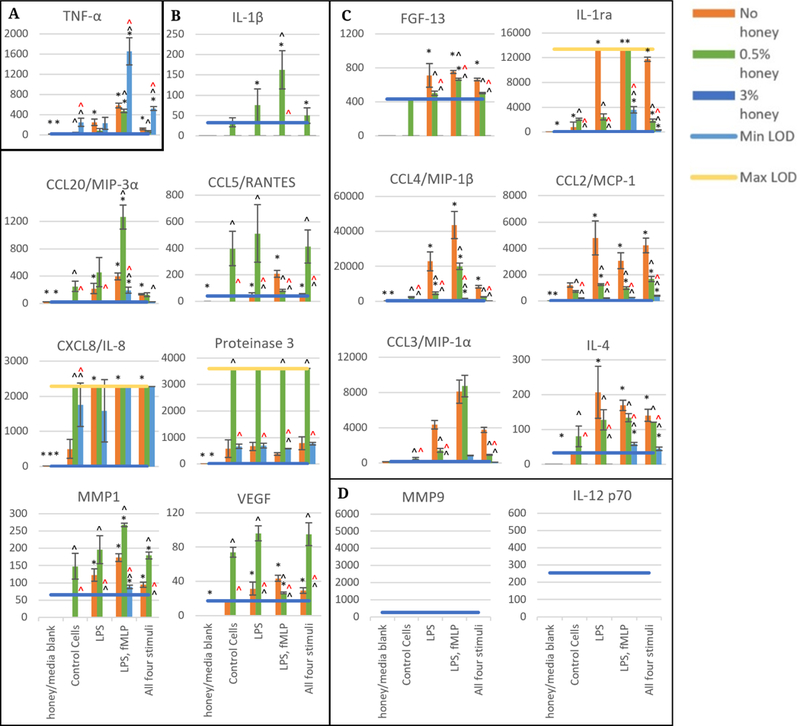
Released levels at 3 hours in the absence of honey or presence of 0.5, and 3% Manuka honey. Scale bars are mean ± standard deviation. “*” indicates a statistically significant difference from the non-stimulated control at that respective honey level, “^” indicates a statistically significant difference from the non-honey group of that stimulation type, and “^” indicates a statistically significant difference from the 0.5% honey group of that stimulation type. A Kruskal-Wallis test was used with a Bonferroni adjustment for multiple comparisons to establish significant differences between stimulation types at each honey level and between honey levels at each stimulation type (α = 0.05). Analytes are grouped by trend (**A**–**D**). *N* = 3.

Figure 2 contains the release data at 3 hours in the presence of 0.5% and 3% Manuka honey, grouped by trend. Figure 2A displays the TNF-α release data and indicates that TNF-α release was increased in the presence of 3% honey relative to 0 and 0.5%. Release at all concentrations of honey was maximal in the LPS, fMLP group. TNF-α was the only analyte measured whose release was increased by 3% honey.

Figure 2B contains the release results for IL-1β, CCL20/MIP-3α, CCL5/RANTES, CXCL8/IL-8, Proteinase 3, MMP-1, and VEGF. These analytes had their release increased in all or most stimulation groups in the presence of 0.5% honey. The LPS, fMLP group had the highest release of IL-1β, CCL20/MIP-3β, and MMP-1 in both the absence and the presence of honey, while 0.5% honey reduced the release of CCL5/RANTES and VEGF in this group. Release of CXCL8/IL-8 was above the Max LOD in most groups, making it difficult to discern trends for this analyte. However, CXCL8/IL-8 release was increased in the control cells in the presence of 0.5% honey. Proteinase 3 release was above the Max LOD for all 0.5% honey samples, and was higher than the 0% honey samples in all stimulation groups except LPS, in which this difference was not statistically significant. 3% honey caused release of IL-1β, CCL20/MIP-3α, CCL5/RANTES, MMP-1, and VEGF to be minimal, but caused no change from the non-honey sample in Proteinase 3 release. IL-8 release increased in the presence of 3% honey in the control cells, but had a non-significant decreasing trend in the LPS-treated cells relative to the non-honey samples. In general, these results reveal that 0.5% honey increased release while 3% honey decreased release for the analytes in Figure 2B. The release data for FGF-13, IL-1ra, CCL4/MIP-1β, CCL2/MCP-1, CCL3/MIP-1α, and IL-4 are displayed in Figure 2C. In general, 0.5% honey either had no effect or caused a decrease in the release of these analytes relative to the non-honey samples, and 3% honey caused release to become negligible. The LPS, fMLP group had the highest release of CCL4/MIP-1β and CCL3/MIP-1α at 0 and 0.5% honey, and also caused release of IL-1ra above Max LOD. In contrast, the FGF-13, CCL2/MCP-1, and IL-4 release data indicate no discernable trend between stimulation groups, but had a general decrease in release as honey concentration increases. Figure 2D indicates that there was no release above the Min LOD for MMP-9 or IL-12 p70 at this 3-hour timepoint.

**Figure 3.**
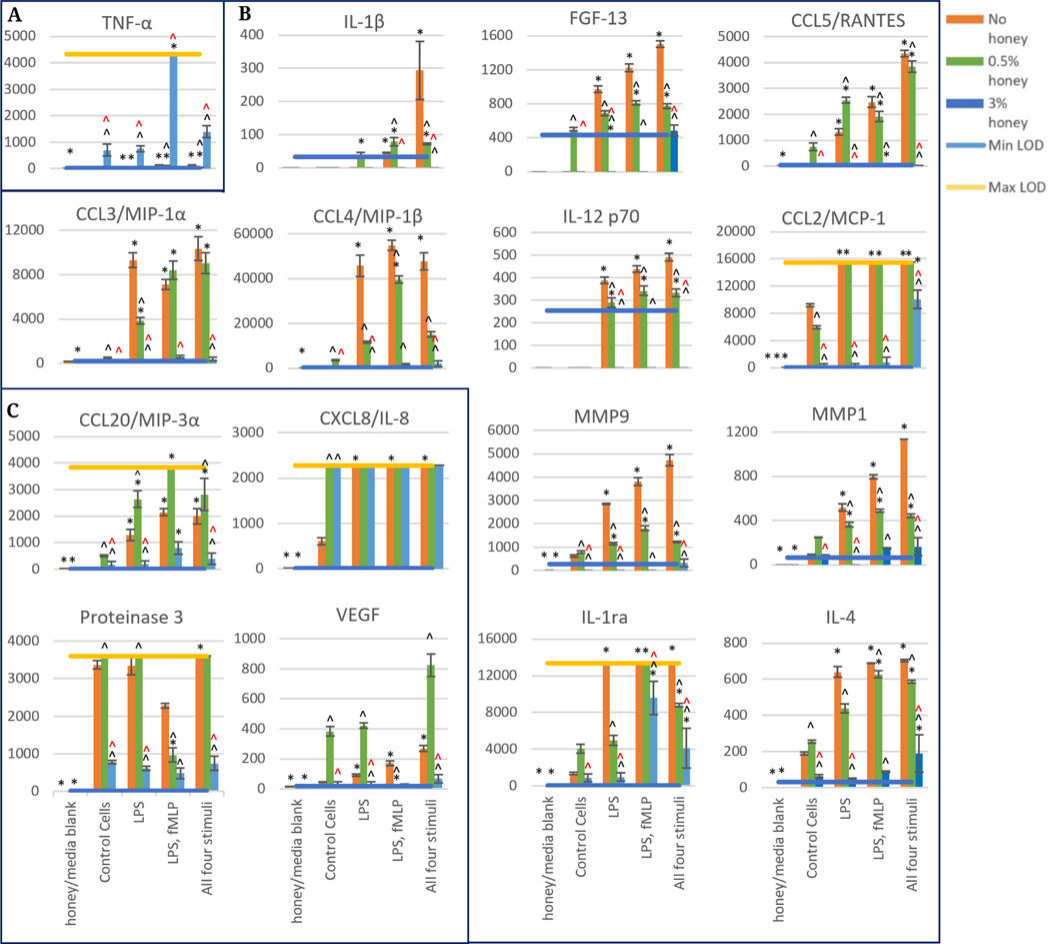
Released levels at 24 hours in the absence of honey or presence of 0.5, and 3% Manuka honey. Scale bars are mean ± standard deviation. “*” indicates a statistically significant difference from the non-stimulated control at that respective honey level, “^” indicates a statistically significant difference from the non-honey group of that stimulation type, and “^” indicates a statistically significant difference from the 0.5% honey group of that stimulation type. A Kruskal-Wallis test was used with a Bonferroni adjustment for multiple comparisons to establish significant differences between stimulation types at each honey level and between honey levels at each stimulation type (α = 0.05). Analytes are grouped by trend (**A**–**C**). *N* = 3.

Figure 3 displays the 24-hour release data for each stimulation group at 0, 0.5, and 3% honey. Similar to the results shown in the 3-hour data, the TNF-α results are grouped by themselves in Figure 3A and show an increase in all stimulation groups in the presence of 3% honey. The greatest release was observed in the LPS, fMLP group, which exceeded the Max LOD. As in the 3-hour results, TNF-α was the only analyte whose release was increased in the presence of 3% honey in all stimulation groups.

Figure 3B contains the release data for IL-1β, FGF-13, CCL5/RANTES, CCL3/MIP-1α, CCL4/MIP-1β, IL-12 p70, CCL2/MCP-1, MMP-9, MMP-1, IL-1ra, and IL-4. These analytes did not increase in most or all stimulation groups at the 0.5% honey level, and many were decreased by this concentration of honey. There was a trend of increasing release between the control cells and the groups stimulated with LPS, LPS and fMLP, and all four stimuli in the IL-1β, FGF-13, CCL5/RANTES, IL-12 p70, MMP-9, and MMP-1 results at 0% honey. However, 0.5% honey obscured this trend for all of these analytes. 3% honey reduced release of these analytes to minimal with only a few exceptions: the all four stimuli group in the CCL2/MCP-1 results, the LPS, fMLP and all four stimuli group in the IL-1ra results, and the all four stimuli group in the IL-4 results. Even in these few exception groups, however, release at 3% honey was lower than the release at the 0 and 0.5% honey levels.

In Figure 3C, the release results for CCL20/MIP-3α, CXCL8/IL-8, Proteinase 3, and VEGF are displayed. These analytes had release results in one or more groups that were increased in the presence of 0.5% honey relative to the 0% honey release levels. 0.5% honey increased the release of CCL20/MIP-3α in the control cells and all stimulation groups. In the CXCL8/IL-8 release results, almost all of the readings were above the Max LOD, making it difficult to draw conclusions about the effect of honey on the release of this analyte. However, it can be observed that release was increased by both 0.5 and 3% honey in the control cells group. Proteinase 3 and VEGF release were increased by 0.5% honey in all groups except the LPS, fMLP group, where release was attenuated by this honey concentration. It is unknown why this trend of increasing release at 0.5% honey would be reversed in the LPS, fMLP group, and this effect is deserving of further study. Release of CCL20/MIP-3α, Proteinase 3, and VEGF was attenuated in the presence of 3% honey in all groups. However, release of CXCL8/IL-8 was increased by 3% honey in the control cells. As CXCL8/IL-8 release at all honey concentrations exceeds the Max LOD in the non-control groups, this trend cannot be confirmed or refuted in these groups.

**Figure 4.**
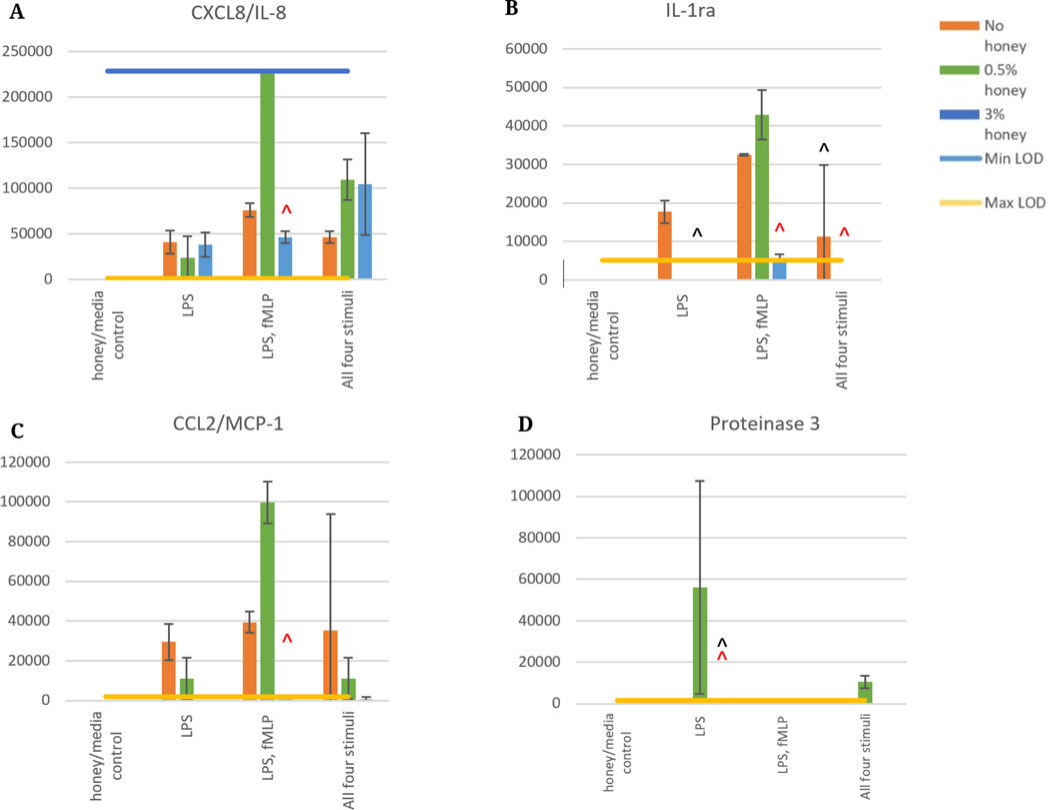
Released levels of CXCL8/IL-8 (**A**), IL-1ra (**B**), CCL2/MCP-1 (**C**), and Proteinase 3 (**D**) at 24 hours in the absence of honey or presence of 0.5, and 3% Manuka honey, measured using a dilution factor of 200 to assay levels above the Max LOD of the earlier assay. Values are expressed as mean ± standard deviation. “*” indicates a statistically significant difference from the non-stimulated control at that respective honey level, “^” indicates a statistically significant difference from the non-honey group of that stimulation type, and “^” indicates a statistically significant difference from the 0.5% honey group of that stimulation type. A Kruskal-Wallis test was used with a Bonferroni adjustment for multiple comparisons to establish significant differences between stimulation types at each honey level and between honey levels at each stimulation type (α = 0.05). *N* = 3.

Figure 4 contains four analytes whose concentrations were above their respective Max LOD values of the assay when originally measured using a dilution factor of 2. This figure displays the values of select 24-hour samples measured utilizing a dilution factor of 200 to extend the range of the assay. The CXCL8/IL-8 results shown in Figure 4A indicate that 0.5% honey increases release in the LPS, fMLP group and causes a non-significant increasing trend in the all four stimuli groups. 3% honey had no effect on release in the LPS group, caused a decrease in the release of the LPS, fMLP group, and appeared to non-significantly increase release in the all four stimuli group although this result had a high degree of variance. Figure 4B indicates that 0.5% honey caused a non-significant increase in IL-1ra release in the LPS, fMLP group but lowered release in the LPS and all four stimuli groups below the Min LOD. 3% honey lowered IL-1ra release in all groups. In Figure 4C, CCL2/MCP-1 release was non-significantly increased in the LPS, fMLP group by 0.5% honey, while release in the other two groups was decreased by 0.5% honey. 3% honey caused IL-1ra release to be minimal in all stimulation groups. Figure 4D displays the Proteinase 3 release data, indicating that release had a non-significant increasing trend caused by 0.5% honey in the groups stimulated by LPS and all four stimuli, but minimal release in all groups in the presence of 3% honey.

It should be noted that the non-honey results in Figure 4D are below the Proteinase 3 release levels displayed for the non-diluted samples in Figure 3, indicating a discrepancy in the values measured between these two experimental runs. This discrepancy could be due to the degradation of the supernatant samples, as they went through a freeze/thaw cycle between the two assay runs. However, it can be seen in the LPS group that the relationship between the 0.5% honey and the non-honey groups is the same, that is, the 0.5% honey group is higher than the non-honey group on both data sets. The results of all parts of this figure have a high degree of variability, which is likely due to the large dilution factor. Nevertheless, the results suggest a trend of greater release of IL-8, IL-1ra, and MCP-1 in the LPS, fMLP group relative to the LPS and LPS, fMLP, IFN-γ, GM-CSF groups. This trend is increased in the presence of 0.5% honey. In contrast, Proteinase 3 trended towards much higher levels in the LPS and LPS, fMLP, IFN-γ, GM-CSF groups with 0.5% honey than in the other stimulation groups. The presence of 3% honey caused a non-significant decreasing trend of IL-1ra and MCP-1 release in all groups. Although the Proteinase 3 release values of the 3% honey samples are below the Min LOD in this figure, Figure 3 indicates that 3% honey decreases Proteinase 3 release in all groups. As observed in Figure 3, however, 3% honey has no significant effect on IL-8 release in any group.

## DISCUSSION

The clinical usage of Manuka honey typically involves the topical application of non-diluted Manuka honey to the wound site [32,34–36,59,60]. While variations of this method have demonstrated effectiveness for *in vivo* wound healing, *in vitro* testing has indicated that Manuka honey concentrations of 5% v/v or above are cytotoxic [38,45]. This cytotoxic effect has been verified *in vivo* in chinchilla ears and ovine frontal sinuses [40,61]. It is likely that when applied to a wound, Manuka honey creates a “zone of death” at the surface which kills bacteria and native human cells alike. As the honey diffuses deeper into the wound environment, it becomes more dilute. However, the majority of implanted tissue engineering devices will not be open to the surface and will not be able to slough off dead cells and excess honey. As such, tissue engineering efforts have focused on incorporating Manuka honey into templates that can release it into the template interior or surrounding tissue at levels below the cytotoxic limit [60–67]. The 0.5% and 3% v/v levels of honey used in this study are similar to the levels of honey likely to be encountered by a neutrophil entering a wound bed or interacting with a honey-laden tissue template. As the implantation of such a template necessitates the creation of a wound, infiltrating neutrophils will encounter an inflammatory environment which has been modeled in this study using LPS, fMLP, IFN-γ, GM-CSF, and various combinations of these stimuli. The effect of Manuka honey on neutrophils within inflammatory environments is highly relevant to the modulation of tissue-template interactions and the desired resolution of inflammation and induction of healing and regeneration.

The cytokine release results indicate that of the stimuli tested, LPS is the main driver of the release of the majority of the analytes tested, including both inflammatory and anti-inflammatory signals. When combined with LPS, fMLP had synergistic effects on release for most analytes. In contrast, the IFN-γ, LPS group only caused a non-statistically-significant increase in the release of CCL3/MIP-1α, CCL4/MIP-1β, CCL20/MIP-3α, MMP-1, and VEGF at 24 hours relative to LPS alone. For some analytes, release was decreased by the combination of all four stimuli relative to the other stimulation groups. These analytes include TNF-α (24-hour timepoint), CCL3/MIP-1α (3-hour timepoint), CCL4/MIP-1β (3-hour timepoint), and CCL20/MIP-3α (3-hour timepoint). For other analytes, though, release was increased by the combination of all four stimuli. These groups include IL-1β (24-hour timepoint), CCL5/RANTES (24-hour timepoint), IL-12 p70 (24-hour timepoint), MMP-9, MMP-1, Proteinase 3 (24-hour timepoint), VEGF (24-hour timepoint), and IL-4 (24-hour timepoint). These results are to be expected given that different signaling receptors and pathways are activated by LPS, fMLP, IFN-γ, and GM-CSF as discussed in the introduction section. The activation of these mechanisms causes release to be increased over the levels observed when only one or two of these mechanisms are activated.

The introduction of Manuka honey into this system caused a change in release that was highly dependent upon the concentration of honey present. 0.5% honey caused a decrease in all or most stimulation groups in the release of most analytes. In contrast, the release of IL-1β, CCL20/MIP-3α, CCL5/RANTES, Proteinase 3, VEGF, CXCL8/IL-8, and CCL2/MCP-1 was increased at one or both timepoints by the presence of 0.5% honey. 3% honey caused a decrease in the release of all analytes at all timepoints except TNF-α and CXCL8/IL-8. As TNF-α and CXCL8/IL-8 are pro-inflammatory signals, these results suggest that 3% honey has a pro-inflammatory effect in this model.

A similar study by Tonks *et al.* corroborates this pro-inflammatory effect *in vitro* in monocytes. In this study, the culture media of an MM6 cell line monocyte model and human peripheral blood monocytes was supplemented with 1% v/v of several honey types, including Manuka honey, and the release of IL-1, IL-6, and TNF-α was measured over 24 hours. All honey types tested, including Manuka honey, caused a significant increase in IL-6, IL-1, and TNF-α release in both the MM6 cells and the peripheral blood monocytes over control cells with no honey or artificial sugar syrup [68]. The increase in the release of these cytokines (particularly the ~8-fold increase in the pro-inflammatory TNF-α) suggests a pro-inflammatory effect of the honey similar to that suggested by the results of 3% honey in this study. However, the results from multiple *in vivo* models indicate an opposite, anti-inflammatory effect of Manuka honey [29,31,69]. In particular, a study by Zhodi *et al.* utilized a Gelam honey-loaded (Gelam is a variety of honey similar to Manuka) PVP/PEG hydrogel in a rat burn wound model, and found that the honey hydrogels caused a significant reduction in the expression of IL-1α, IL-1β, and IL-6 expression within the wound site [70]. This expression was measured from total RNA taken from the wound, so it is unknown how much of this RNA is from neutrophils, macrophages, fibroblasts, etc., but nevertheless these results point to a general inflammation-reducing effect of the honey *in vivo*. Unfortunately, TNF-α expression in the wound was not measured, so it is unknown whether the honey increases its *in vivo* expression. The release of honey from the hydrogel was not quantified, so it is also unknown what the level of honey is in the wound at each timepoint. Nevertheless, this study is currently the best measure of honey’s *in vivo* effect on cytokine production within a wound. In a 2014 review paper, Majtan theorized that honey’s pro/anti-inflammatory effects are dependent on the state of the wound environment, upregulating inflammation in acute wounds while downregulating inflammation in chronically-inflamed wounds. While there is some evidence to suggest this effect, as detailed in the review, more investigation is needed to fully explore and verify this claim [71].

Another factor to consider is the number of neutrophils that arrive at the wound site. A 2011 study by Leong et al. used arachidonic acid to create inflammation in a mouse model, and found that the topical application of Manuka honey reduced the infiltration of neutrophils into the wound site by around a factor of 2 [72]. We have also previously reported that Manuka honey reduces dHL-60 chemotaxis to fMLP [45]. As such, while our results indicate that 3% honey increases TNF-α release at both 3 and 24 hours, it is possible that the overall drop in neutrophil number, coupled with the decrease in release of all other factors measured in this study except IL-8, causes the inflammation decrease observed *in vivo*. The results at 0.5% honey are more confounding, with no clear pattern between the effect of the analyte (e.g., pro-inflammatory, ECM-degrading, angiogenic, or anti-inflammatory) and its change in release levels relative to non-honey controls. Although TNF-α, CCL3/MIP-1α, and CCL4/MIP-1β were decreased at both timepoints in most or all samples, CCL20/MIP-3α, CCL5/RANTES, and CXCL8/IL-8 all were increased in one or more sample groups by this concentration of honey. Thus, no definitive conclusion can be drawn from these results regarding the effect of 0.5% honey on the regulation of inflammation by neutrophils. As shown above, both concentrations of Manuka honey decreased MMP9 and MMP1 production at the 24-hour timepoint. As excessive amounts of MMPs have been implicated in ongoing tissue damage during chronic inflammation, this MMP regulating effect of the honey may be a key component to the reduction of tissue damage [73,74]. Ultimately, an *in vivo* study in which different concentrations of honey are applied to a wound model will be necessary to ascertain the effect of low (below 1% v/v) honey concentrations on the resolution of inflammation and activation of the healing response.

Although the mechanisms by which Manuka honey causes these cellular effects are not fully known, it is theorized that the phenolic components of honey, which include known bioactive molecules like pinobanksin and pinocembrin, can cross the cellular membrane [47,75–77]. Under this theory, these phenolic molecules neutralize free radicals within the cell and trigger AMPK phosphorylation, increasing the expression of antioxidant enzymes and modifying numerous intracellular pathways [20,78]. While this proposed mechanism of action is plausible, more work is required to fully validate it and elucidate other possible methods by which Manuka honey modifies neutrophil behavior. It should be noted that Manuka honey has previously been shown to contain trace amounts of LPS, although these levels are below the minimum needed to stimulate most neutrophil inflammatory behaviors [68,79]. Nevertheless, this LPS content may play a role in the cytokine profiles reported in this paper, as one bioactive component among many. As Manuka honey is a natural product, there is no practical way to eliminate this LPS component, nor is it necessary. The clinical and *in vitro* evidence described in the introduction demonstrates that Manuka honey has valuable pro-healing effects in spite of its trace LPS content.

## CONCLUSIONS

The results of this study indicate that Manuka honey drastically changes the release of cytokines, chemokines, and matrix-degrading enzymes from the dHL-60 neutrophil model. This change is highly dependent on the concentration of honey present and the inflammatory preconditioning of the cells. These findings suggest that honey-releasing tissue engineering templates could elicit a variety of effects with regards to neutrophil behavior and inflammation resolution depending on the release profile of the honey from the template. Future work will focus on the effect of honey on dHL-60s under anti-inflammatory conditions (*i.e*., TGF-β, IL-4, IL-13) and the *in vivo* modulation of inflammation by honey-containing templates with different honey loads and release profiles.

## Supplementary Material

1

2

3

4

5

## Footnotes

SUPPLEMENTARY MATERIALS The following supplementary materials are available online at https://doi.org/10.20900/mo.20190005, Raw data: Experiment 1—no honey, Experiment 2—0.5 percent honey, Experiment 3—3 percent honey, Raw RANTES redouts; Supplementary tables: Individual *p* values for multiple comparisons.

## ABBREVIATIONS

AMPK: 5’ AMP-activated protein kinase
ATCC: American Type Culture Collection
CCL: chemokine (C-C motif) ligand
CD: classification determinant or cluster of differentiation
CXCL: chemokine (C-X-C motif) ligand
DAPI: 6-diamidino-2-phenylindole
dHL-60: differentiated HL-60
DMSO: dimethylsulfoxide
ECM: extracellular matrix
FBS: fetal bovine serum
FGF: fibroblast growth factor
fMLP: *N*-formylmethionine-leucyl-phenylalanine
GCPR: G-coupled protein receptor
GM-CSF: granulocyte-macrophage colony-stimulating factor
HPLC-MS: high performance liquid chromatography–mass spectrometry
IFN-γ: interferon-γ
IL: interleukin
IL-1ra: interleukin 1 receptor antagonist
JAK: janus kinase
LPS: lipopolysaccharide
MCP: monocyte chemoattractant protein
MIP: macrophage inflammatory protein
MMP: matrix metalloproteinase
MRSA: methicillin-resistant *Staphylococcus aureus*
MyD88: myeloid differentiation primary response 88
NADPH: nicotinamide adenine dinucleotide phosphate
NF: nuclear factor
NK cell: natural killer cell
Pen/Strep: penicillin/streptomycin
PI3: phosphoinositide 3
PLC: phospholipase C
PVP: polyvinylpyrrolidone
PEG: polyethylene glycol
RANTES: regulation on activation normal T cell expressed and secreted
RNA: ribonucleic acid
RPMI: Roswell Park Memorial Institute (medium)
SHC: src homology 2 domain-containing
STAT: signal transducer and activator of transcription proteins
TGF: transforming growth factor
TLR: toll-like receptor
TNF: tumor necrosis factor
VEGF: vascular endothelial growth factor
