# Supplementary material for: The Effect of Manuka Honey on dHL-60 Cytokine, Chemokine, and Matrix-Degrading Enzyme Release under Inflammatory Conditions
Source: Med One. Author manuscript; Available in PMC 2019 Jun 26. (PMC6594701; doi:10.20900/mo.20190005)
Supplement: 5 [file NIHMS1026110-supplement-5.pdf]

			Multiple Comparisons							
		**Kruskal-Wallis**	**Control Cells**	**fMLP**	**GM-CSF**	**IFN-γ**	**LPS**	**IFN-γ, LPS**	**LPS, fMLP**	**All four stimuli**
Box A	TNF-α	0.0003	Ref	1	1	1	<0.0001	<0.0001	<0.0001	<0.0001
Box B	IL-1β	0.0818	Ref	1	1	1	0.1355	1	1	1
Box C	MMP-1	0.0009	Ref	1	1	1	<0.0001	0.0001	<0.0001	0.0012
	CCL5/RANTES	0.0016	Ref	1	1	1	0.0020	0.0261	<0.0001	0.0010
	CCL20/MIP-3α	0.0003	Ref	1	1	1	<0.0001	<0.0001	<0.0001	<0.0001
	MMP-9	1	Ref	-	-	-	-	-	-	-
	IL-12 p70	0.4433	Ref	1	1	1	1	1	1	1
	VEGF	0.0004	Ref	0.3320	0.0007	1	<0.0001	0.0001	<0.0001	<0.0001
Box D	CCL3/MIP-1α	0.0008	Ref	1	1	1	<0.0001	<0.0001	<0.0001	0.0008
	CCL4/MIP-1β	0.0003	Ref	0.0002	1	1	<0.0001	<0.0001	<0.0001	<0.0001
	FGF-13	0.0006	Ref	1	1	1	<0.0001	<0.0001	<0.0001	<0.0001
Box E	IL-1ra	0.0005	Ref	1	1	1	<0.0001	<0.0001	<0.0001	<0.0001
	CCL2/MCP-1	0.0005	Ref	0.0028	0.0010	1	<0.0001	<0.0001	<0.0001	<0.0001
	Proteinase 3	0.0196	Ref	1	1	1	1	1	1	1
	CXCL8/IL-8	0.0003	Ref	<0.0001	<0.0001	0.3740	<0.0001	<0.0001	<0.0001	<0.0001
	IL-4	0.0007	Ref	1	1	1	<0.0001	<0.0001	0.0001	0.0425

Table S1. P-values for released levels with no honey at 3 hours compared to nonstimulated control cells. A Kruskal-Wallis test was used with a Bonferroni adjustment for multiple comparisons (α=0.05).

			Multiple Comparisons							
		**Kruskal-Wallis**	**Control Cells**	**fMLP**	**GM-CSF**	**IFN-γ**	**LPS**	**IFN-γ, LPS**	**LPS, fMLP**	**All four stimuli**
Box A	TNF-α	0.0003	Ref	1	1	1	<0.0001	<0.0001	<0.0001	<0.0001
Box B	IL-1β	0.0026	Ref	1	1	1	1	1	0.0020	<0.0001
Box C	MMP-1	0.0004	Ref	1	0.0104	1	<0.0001	<0.0001	<0.0001	<0.0001
	CCL5/RANTES	0.0003	Ref	1	<0.0001	1	<0.0001	<0.0001	<0.0001	<0.0001
	CCL20/MIP-3α	0.0006	Ref	1	1	1	<0.0001	<0.0001	<0.0001	<0.0001
	MMP-9	0.0003	Ref	0.0029	0.2412	1	<0.0001	<0.0001	<0.0001	<0.0001
	IL-12 p70	0.0004	Ref	1	1	1	<0.0001	<0.0001	<0.0001	<0.0001
	VEGF	0.0005	Ref	1	<0.0001	0.2371	0.0037	<0.0001	<0.0001	<0.0001
Box D	CCL3/MIP-1α	0.0009	Ref	1	1	1	0.0001	<0.0001	0.0022	<0.0001
	CCL4/MIP-1β	0.0003	Ref	1	0.0013	0.4361	<0.0001	<0.0001	<0.0001	<0.0001
	FGF-13	0.0006	Ref	1	1	1	0.0001	<0.0001	<0.0001	<0.0001
Box E	IL-1ra	0.0003	Ref	0.0529	<0.0001	0.9170	<0.0001	<0.0001	<0.0001	<0.0001
	CCL2/MCP-1	0.0004	Ref	1	<0.0001	0.0222	<0.0001	<0.0001	<0.0001	<0.0001
	Proteinase 3	0.0065	Ref	1	0.9395	1	1	1	1	0.9395
	CXCL8/IL-8	0.0007	Ref	1	<0.0001	1	<0.0001	<0.0001	<0.0001	<0.0001
	IL-4	0.0003	Ref	1	<0.0001	0.5630	<0.0001	<0.0001	<0.0001	<0.0001

Table S2. P-values for released levels with no honey at 24 hours compared to nonstimulated control cells. A Kruskal-Wallis test was used with a Bonferroni adjustment for multiple comparisons (α=0.05).

		Control Cells	fMLP	GM-CSF	IFN-γ	LPS	IFN-γ, LPS	LPS, fMLP	All four stimuli
Box A	TNF-α	1	1	1	1	0.0809	0.0809	0.0809	0.3827
Box B	IL-1β	1	1	1	1	0.3537	0.5050	0.0636	0.0765
Box C	MMP-1	0.0469	0.6428	0.0765	0.0593	0.0809	0.0809	0.0765	0.0809
	CCL5/RANTES	1	0.5050	0.0765	0.5050	0.0809	0.0765	0.0809	0.0809
	CCL20/MIP-3α	1	1	0.5050	0.5050	0.0809	0.0809	0.0809	0.0809
	MMP-9	0.0636	0.0636	0.0636	0.0636	0.0636	0.0636	0.0636	0.0636
	IL-12 p70	1	1	0.5050	1	0.0722	0.0593	0.0593	0.0636
	VEGF	0.0809	0.0809	0.0809	0.0809	0.0809	0.0765	0.0765	0.0809
Box D	CCL3/MIP-1α	1	0.5050	1	1	0.0809	0.0809	0.6625	0.0809
	CCL4/MIP-1β	0.0809	0.3827	0.0765	0.0765	0.0809	0.0809	0.0809	0.0809
	FGF-13	1	1	0.5050	1	0.0809	0.0809	0.0765	0.0809
Box E	IL-1ra	0.6625	0.3827	0.0809	0.0809	0.0765	0.0809	0.0636	0.0809
	CCL2/MCP-1	0.0809	0.0809	0.0636	0.0809	0.0636	0.0636	0.0636	0.0636
	Proteinase 3	0.0809	0.0809	0.0636	0.0809	0.0809	0.0809	0.0809	0.0636
	CXCL8/IL-8	0.6625	0.0636	0.0636	0.0809	1	1	1	1
	IL-4	0.0593	0.0765	0.0809	0.0636	0.0809	0.0809	0.0809	0.0809

Table S3. P-values for released levels with no honey at 3 hours compared to 24 hours. Wilcoxon rank sum test was used to test differences between timepoints for each stimulation type (α=0.05).

			Multiple Comparisons			
		Kruskal-Wallis	Control Cells	LPS	LPS, fMLP	All four stimuli
Box A	TNF-α	0.0046	Ref	1	<0.0001	1
Box B	IL-1β	0.0627	Ref	1	0.6226	1
	CCL20/MIP-3α	0.0035	Ref	0.5273	0.0043	0.5273
	CCL5/RANTES	0.0047	Ref	0.4623	0.4623	1
	CXCL8/IL-8	0.0018	Ref	<0.0001	<0.0001	<0.0001
	Proteinase 3	0.0033	Ref	1	1	1
	MMP-1	0.0067	Ref	1	0.0011	0.0011
	VEGF	0.0025	Ref	0.9385	<0.0001	1
Box C	FGF-13	0.0028	Ref	0.4359	0.0011	0.5116
	IL-1ra	0.0029	Ref	1	<0.0001	1
	CCL4/MIP-1β	0.0021	Ref	0.0263	<0.0001	1
	CCL2/MCP-1	0.0023	Ref	<0.0001	0.0327	<0.0001
	CCL3/MIP-1α	0.0023	Ref	0.0083	<0.0001	0.4583
	IL-4	0.0038	Ref	0.3109	0.2003	1
Box D	MMP-9	0.4289	Ref	1	1	1
	IL-12 p70	1	Ref	-	-	-

Table S4. P-values for released levels with 0.5% honey at 3 hours compared to nonstimulated controls. A Kruskal-Wallis test was used with a Bonferroni adjustment for multiple comparisons (α=0.05).

			Multiple Comparisons			
		Kruskal-Wallis	Control Cells	LPS	LPS, fMLP	All four stimuli
Box A	TNF-α	0.0045	Ref	1	0.0081	0.0453
Box B	IL-1β	1	Ref	-	-	-
	CCL20/MIP-3α	0.0026	Ref	1	<0.0001	1
	CCL5/RANTES	0.0027	Ref	1	1	1
	CXCL8/IL-8	0.0095	Ref	1	1	0.6693
	Proteinase 3	0.0243	Ref	1	1	1
	MMP-1	0.0038	Ref	<0.0001	<0.0001	<0.0001
	VEGF	0.0041	Ref	1	0.0001	0.0020
Box C	FGF-13	1	Ref	-	-	-
	IL-1ra	0.0044	Ref	1	<0.0001	0.0387
	CCL4/MIP-1β	0.0026	Ref	1	<0.0001	1
	CCL2/MCP-1	0.0031	Ref	1	0.1925	0.0007
	CCL3/MIP-1α	0.0026	Ref	1	<0.0001	1
	IL-4	0.0037	Ref	1	0.0004	0.0011
Box D	MMP-9	1	Ref	-	-	-
	IL-12 p70	1	Ref	-	-	-

Table S5. P-values for released levels with 3% honey at 3 hours compared to nonstimulated controls. A Kruskal-Wallis test was used with a Bonferroni adjustment for multiple comparisons (α=0.05).

		Across all concentrations (Kruskal-Wallis)				3% compared to 0.5% honey (Multiple comparisons)			
		Control Cells	LPS	LPS, fMLP	All four stimuli	Control Cells	LPS	LPS, fMLP	All four stimuli
Box A	TNF-α	0.0241	0.0650	0.0273	0.0273	0.0123	0.0724	0.0010	0.0010
Box B	IL-1β	0.3679	0.2369	0.0221	0.0986	0.7997	0.4881	0.0002	0.0878
	CCL20/MIP-3α	0.0221	0.0459	0.0273	0.0552	0.0002	0.0140	0.0010	0.0720
	CCL5/RANTES	0.0221	0.0241	0.0241	0.0241	0.0002	0.0003	0.0123	0.0003
	CXCL8/IL-8	0.0273	0.1054	1	1	0.0312	0.1587	-	-
	Proteinase 3	0.0552	0.0605	0.0273	0.0552	0.0285	0.0455	0.0312	0.0720
	MMP-1	0.0204	0.0234	0.0249	0.0241	<0.0001	0.0002	0.0004	0.0003
	VEGF	0.0273	0.0265	0.0257	0.0265	0.0010	0.0008	0.0208	0.0008
Box C	FGF-13	0.1054	0.0241	0.0234	0.0241	0.1587	0.0123	0.0089	0.0123
	IL-1ra	0.0273	0.0265	0.0221	0.0273	0.0010	0.0257	0.0002	0.0312
	CCL4/MIP-1β	0.0241	0.0241	0.0273	0.0241	0.0003	0.0123	0.0312	0.0123
	CCL2/MCP-1	0.0273	0.0273	0.0273	0.0273	0.0312	0.0312	0.0312	0.0312
	CCL3/MIP-1α	0.0221	0.0241	0.0665	0.0241	0.0002	0.0123	0.0569	0.0123
	IL-4	0.0211	0.0257	0.0265	0.0234	<0.0001	0.0208	0.0257	0.0089
Box D	MMP-9	1	0.3679	1	1	-	0.7997	-	-
	IL-12 p70	1	0.3679	1	1	-	1	-	-

Table S6. Part 1 of p-values for released levels between honey concentrations 0%, 0.5%, and 3% at 3 hours. A Kruskal-Wallis test was used with a Bonferroni adjustment for multiple comparisons (α=0.05).

		3% compared to 0% honey (Multiple comparisons)				0.5% compared to 0% honey (Multiple comparisons)			
		**Control Cells**	**LPS**	**LPS, fMLP**	**All four stimuli**	**Control Cells**	**LPS**	**LPS, fMLP**	**All four stimuli**
Box A	TNF-α	0.0003	1	0.0312	0.0312	0.0123	0.0540	0.0312	0.0312
Box B	IL-1β	1	0.4881	1	0.9460	0.7997	1	0.0002	0.3906
	CCL20/MIP-3α	1	0.0709	0.0312	0.0285	0.0002	0.6598	0.0312	1
	CCL5/RANTES	1	0.0123	0.0003	0.0123	0.0002	0.0123	0.0123	0.0123
	CXCL8/IL-8	0.0312	0.1587	-	-	0.0010	1	-	-
	Proteinase 3	1	1	0.0312	1	0.0720	0.0616	0.0010	0.0285
	MMP-1	0.0010	0.0089	0.0163	0.0123	0.0010	0.0089	0.0163	0.0123
	VEGF	0.0312	0.0257	0.0006	0.0257	0.0312	0.0257	0.0208	0.0257
Box C	FGF-13	1	0.0003	0.0002	0.0003	0.1587	0.0123	0.0089	0.0123
	IL-1ra	0.0312	0.0008	0.0002	0.0010	0.0312	0.0257	1	0.0312
	CCL4/MIP-1β	0.0123	0.0003	0.0010	0.0003	0.0123	0.0123	0.0312	0.0123
	CCL2/MCP-1	0.0010	0.0010	0.0010	0.0010	0.0312	0.0312	0.0312	0.0312
	CCL3/MIP-1α	1	0.0003	0.0762	0.0003	0.0002	0.0123	1	0.0123
	IL-4	0.0021	0.0006	0.0008	0.0002	0.0021	0.0208	0.0257	0.0089
Box D	MMP-9	-	1	-	-	-	0.7997	-	-
	IL-12 p70	-	0.7997	-	-	-	0.7997	-	-

Table S7. Part 2 of p-values for released levels between honey concentrations 0%, 0.5%, and 3% at 3 hours. A Kruskal-Wallis test was used with a Bonferroni adjustment for multiple comparisons (α=0.05).

			Multiple Comparisons			
		**Kruskal-Wallis**	**Control Cells**	**LPS**	**LPS, fMLP**	**All four stimuli**
Box A	TNF-α	0.0025	Ref	<0.0001	<0.0001	0.0007
Box B	IL-1β	0.0034	Ref	0.0004	<0.0001	<0.0001
	FGF-13	0.0071	Ref	1	0.0001	0.0032
	CCL5/RANTES	0.0027	Ref	<0.0001	<0.0001	<0.0001
	CCL3/MIP-1α	0.0023	Ref	0.0005	<0.0001	<0.0001
	CCL4/MIP-1β	0.0025	Ref	1	0.0011	0.0871
	IL-12 p70	0.0077	Ref	<0.0001	<0.0001	<0.0001
	CCL2/MCP-1	0.0018	Ref	<0.0001	<0.0001	<0.0001
	MMP-9	0.0022	Ref	<0.0001	<0.0001	<0.0001
	MMP-1	0.0025	Ref	0.0040	<0.0001	<0.0001
	IL-1ra	0.0019	Ref	0.1779	<0.0001	<0.0001
	IL-4	0.0022	Ref	0.4609	<0.0001	<0.0001
Box C	CCL20/MIP-3α	0.0032	Ref	<0.0001	<0.0001	<0.0001
	CXCL8/IL-8	0.0017	Ref	-	-	-
	Proteinase 3	0.0020	Ref	1	<0.0001	1
	VEGF	0.0022	Ref	1	<0.0001	1

Table S8. P-values for released levels with 0.5% honey at 24 hours compared to nonstimulated controls. A Kruskal-Wallis test was used with a Bonferroni adjustment for multiple comparisons (α=0.05).

		Kruskal-Wallis	*Multiple Comparisons* Control Cells	LPS	LPS, fMLP	All four stimuli
Box A	TNF-α	0.0038	Ref	1	0.0118	0.1049
Box B	IL-1β	1	Ref	-	-	-
	FGF-13	0.1775	Ref	1	1	1
	CCL5/RANTES	0.0026	Ref	1	<0.0001	1
	CCL3/MIP-1α	0.0251	Ref	1	0.2229	1
	CCL4/MIP-1β	0.0317	Ref	1	0.0861	0.0706
	IL-12 p70	1	Ref	-	-	-
	CCL2/MCP-1	0.0039	Ref	1	1	<0.0001
	MMP-9	0.0523	Ref	1	1	0.0534
	MMP-1	0.0041	Ref	0.1790	0.4940	0.4326
	IL-1ra	0.0058	Ref	1	0.0006	0.0125
	IL-4	0.0055	Ref	1	1	0.0227
Box C	CCL20/MIP-3α	0.0072	Ref	1	0.0484	0.9404
	CXCL8/IL-8	0.0025	Ref	-	-	-
	Proteinase 3	0.0138	Ref	1	0.3137	1
	VEGF	0.0179	Ref	1	1	0.5182

Table S9. P-values for released levels with 3% honey at 24 hours compared to nonstimulated controls. A Kruskal-Wallis test was used with a Bonferroni adjustment for multiple comparisons (α=0.05).

		Across all concentrations (Kruskal-Wallis)				*3% compared to 0.5% honey (Multiple comparisons)*			
		**Control Cells**	**LPS**	**LPS, fMLP**	**All four stimuli**	**Control Cells**	**LPS**	**LPS, fMLP**	**All four stimuli**
Box A	TNF-α	0.0329	0.0265	0.0439	0.0273	0.0298	0.0008	0.0043	0.0010
Box B	IL-1β	1	0.0625	0.0423	0.0241	-	0.0351	0.0033	0.0123
	FGF-13	0.0347	0.0241	0.0423	0.0273	0.0163	0.0123	0.0841	0.0312
	CCL5/RANTES	0.0221	0.0241	0.0439	0.0241	0.0002	0.0003	0.1029	0.0123
	CCL3/MIP-1α	0.0340	0.0241	0.0685	0.0390	0.0340	0.0123	0.0299	0.0748
	CCL4/MIP-1β	0.0265	0.0265	0.0439	0.0273	0.0008	0.0257	0.1029	0.0312
	IL-12 p70	1	0.0234	0.0407	0.0234	-	0.0089	0.0664	0.0089
	CCL2/MCP-1	0.0273	0.0221	0.0324	0.0221	0.0312	0.0002	0.0001	0.0002
	MMP-9	0.0241	0.0241	0.0423	0.0273	0.0003	0.0123	0.0841	0.0312
	MMP-1	0.0538	0.0241	0.0423	0.0265	0.0266	0.0123	0.0841	0.0257
	IL-1ra	0.0390	0.0241	0.0324	0.0241	0.0070	0.0123	0.0001	0.0123
	IL-4	0.0257	0.0265	0.0423	0.0273	0.0006	0.0257	0.0841	0.0312
Box C	CCL20/MIP-3α	0.0241	0.0273	0.0376	0.0273	0.0123	0.0010	0.0011	0.0010
	CXCL8/IL-8	0.0221	1	1	1	1	-	-	-
	Proteinase 3	0.0241	0.0241	0.0439	0.0221	0.0003	0.0003	0.1029	0.0002
	VEGF	0.0608	0.0273	0.0423	0.0273	0.0369	0.0010	0.0841	0.0010

Table S10. Part 1 of p-values for released levels between honey concentrations 0%, 0.5%, and 3% at 24 hours. A Kruskal-Wallis test was used with a Bonferroni adjustment for multiple comparisons (α=0.05).

		3% compared to 0% honey (Multiple comparisons)				0.5% compared to 0% honey (Multiple comparisons)			
		**Control Cells**	**LPS**	**LPS, fMLP**	**All four stimuli**	**Control Cells**	**LPS**	**LPS, fMLP**	**All four stimuli**
Box A	TNF-α	0.0034	0.0257	0.1029	0.0312	0.2344	0.0257	0.0352	0.0312
Box B	IL-1β	-	1	0.0841	0.0003	-	0.1298	0.0278	0.0123
	FGF-13	0.9890	0.0003	0.0033	0.0010	0.0055	0.0123	0.0278	0.0312
	CCL5/RANTES	1	0.0123	0.0043	0.0003	0.0002	0.0123	0.0352	0.0123
	CCL3/MIP-1α	0.2559	0.0003	0.2334	0.0070	0.0039	0.0123	0.2914	0.2484
	CCL4/MIP-1β	0.0257	0.0008	0.0043	0.0010	0.0257	0.0257	0.0352	0.0312
	IL-12 p70	-	0.0002	0.0024	0.0002	-	0.0089	0.0212	0.0089
	CCL2/MCP-1	0.0010	0.0002	0.0001	0.0002	0.0312	1	1	1
	MMP-9	0.0123	0.0003	0.0033	0.0010	0.0123	0.0123	0.0278	0.0312
	MMP-1	1	0.0003	0.0033	0.0008	0.0677	0.0123	0.0278	0.0257
	IL-1ra	0.2484	0.0003	0.0001	0.0003	0.0748	0.0123	1	0.0123
	IL-4	0.0208	0.0008	0.0033	0.0010	0.0208	0.0257	0.0278	0.0312
Box C	CCL20/MIP-3α	0.0123	0.0312	0.0352	0.0312	0.0003	0.0312	0.0104	0.0312
	CXCL8/IL-8	0.0002	-	-	-	0.0002	-	-	-
	Proteinase 3	0.0123	0.0123	0.0043	0.0002	0.0123	0.0123	0.0352	1
	VEGF	1	0.0312	0.0841	0.0312	0.0901	0.0312	0.0030	0.0312

Table S11. Part 2 of p-values for released levels between honey concentrations 0%, 0.5%, and 3% at 24 hours. A Kruskal-Wallis test was used with a Bonferroni adjustment for multiple comparisons (α=0.05).

		Kruskal-Wallis (across LPS; LPS, fMLP; and all four stimuli)		
		**0% honey**	**0.5% honey**	**3% honey**
A	CXCL8/IL-8	0.0687	0.0439	0.1767
B	IL-1ra	0.0687	0.0783	0.0390
C	CCL2/MCP-1	0.1073	0.0439	0.0556
D	Proteinase 3	0.0654	0.0439	0.1968

Table S12. P-values for released levels of CXCL8/IL-8, IL-1ra, CCL2/MCP-1, and Proteinase 3 compared to nonstimulated control cells at 24 hours in the presence of 0, 0.5, and 3% Manuka honey, measured using a dilution factor of 200 to assay levels above the Max LOD of the earlier assay.

		Across all concentrations (Kruskal-Wallis)			*3% compared to 0.5% honey (Multiple comparisons)*		
		**LPS**	**LPS, fMLP**	**All four stimuli**	**LPS**	**LPS, fMLP**	**All four stimuli**
A	CXCL8/IL-8	0.7897	0.0439	0.1335	1	0.0040	1
B	IL-1ra	0.0439	0.0439	0.0439	0.1029	0.0040	0.0352
C	CCL2/MCP-1	0.0541	0.0423	0.0742	0.1080	0.0030	0.0450
D	Proteinase 3	0.0423	0.0984	0.0766	0.0033	0.1209	0.0898

Table S13. Part 1 of p-values for released levels between honey concentrations 0%, 0.5%, and 3% at 24 hours for released levels of CXCL8/IL-8, IL-1ra, CCL2/MCP-1, and Proteinase 3, measured using a dilution factor of 200 to assay levels above the Max LOD of the earlier assay. A Kruskal-Wallis test was used with a Bonferroni adjustment for multiple comparisons (α=0.05).

		3% compared to 0% honey (Multiple comparisons)			*0.5% compared to 0% honey (Multiple comparisons)*		
		**LPS**	**LPS, fMLP**	**All four stimuli**	**LPS**	**LPS, fMLP**	**All four stimuli**
A	CXCL8/IL-8	1	0.1029	0.1811	1	0.1029	0.2304
B	IL-1ra	0.0040	0.1029	0.1029	0.1029	0.1029	0.0043
C	CCL2/MCP-1	0.1029	0.0841	1	0.5241	0.0841	0.1404
D	Proteinase 3	0.0278	0.1498	1	0.0841	1	0.0645

Table S14. Part 2 of p-values for released levels between honey concentrations 0%, 0.5%, and 3% at 24 hours for released levels of CXCL8/IL-8, IL-1ra, CCL2/MCP-1, and Proteinase 3, measured using a dilution factor of 200 to assay levels above the Max LOD of the earlier assay. A Kruskal-Wallis test was used with a Bonferroni adjustment for multiple comparisons (α=0.05).
